# Effect of atorvastatin on C-reactive protein and benefits for cardiovascular disease in patients with type 2 diabetes: analyses from the Collaborative Atorvastatin Diabetes Trial

**DOI:** 10.1007/s00125-015-3586-8

**Published:** 2015-04-22

**Authors:** Sabita S. Soedamah-Muthu, Shona J. Livingstone, Valentine Charlton-Menys, D. John Betteridge, Graham A. Hitman, H. Andrew W. Neil, Weihang Bao, David A. DeMicco, Gregory M. Preston, John H. Fuller, Coen D. A. Stehouwer, Casper G. Schalkwijk, Paul N. Durrington, Helen M. Colhoun, on behalf of the CARDS Investigators

**Affiliations:** Division of Human Nutrition, Wageningen University, Bomenweg 2, P.O. Box 8129, 6700 EV Wageningen, the Netherlands; Division of Medicine and Therapeutics, Ninewells Hospital and Medical School, Dundee, UK; University of Manchester Cardiovascular Research Group, Manchester, UK; Department of Medicine, Jules Thorne Institute, The Middlesex Hospital, London, UK; The Blizard Institute, Barts and The London School of Medicine, Queen Mary University of London, London, UK; Division of Public Health and Primary Health Care, University of Oxford, Oxford, UK; Pfizer Inc, New York, NY USA; Department of Epidemiology and Public Health, University College London, London, UK; Department of Internal Medicine, Laboratory for Metabolism and Vascular Medicine, Maastricht University Medical Centre (MUMC), Maastricht, the Netherlands; Cardiovascular Research Institute Maastricht (CARIM), Maastricht University Medical Centre, Maastricht, the Netherlands

**Keywords:** Atorvastatin, Cardiovascular disease, CARDS, C-reactive protein, LDL-cholesterol, Statins, Type 2 diabetes

## Abstract

**Aims/hypothesis:**

We investigated whether atorvastatin 10 mg daily lowered C-reactive protein (CRP) and whether the effects of atorvastatin on cardiovascular disease (CVD) varied by achieved levels of CRP and LDL-cholesterol.

**Methods:**

CRP levels were measured at baseline and 1 year after randomisation to atorvastatin in 2,322 patients with type 2 diabetes (40–75 years, 69% males) in a secondary analysis of the Collaborative Atorvastatin Diabetes Study, a randomised placebo-controlled trial. We used Cox regression models to test the effects on subsequent CVD events (*n* = 147) of CRP and LDL-cholesterol lowering at 1 year.

**Results:**

After 1 year, the atorvastatin arm showed a net CRP lowering of 32% (95% CI −40%, −22%) compared with placebo. The CRP response was highly variable, with 45% of those on atorvastatin having no decrease in CRP (median [interquartile range, IQR] per cent change −9.8% [−57%, 115%]). The LDL-cholesterol response was less variable, with a median (IQR) within-person per cent change of −41% (−51%, −31%). Baseline CRP did not predict CVD over 3.8 years of follow-up (HR_per SD log_ 0.89 [95% CI 0.75, 1.06]), whereas baseline LDL-cholesterol predicted CVD (HR_per SD_ 1.21 [95% CI 1.02, 1.44]), as did on-treatment LDL-cholesterol. There was no significant difference in the reduction in CVD by atorvastatin, with above median (HR 0.57) or below median (HR 0.52) change in CRP or change in LDL-cholesterol (HR 0.61 vs 0.50).

**Conclusions/interpretation:**

CRP was not a strong predictor of CVD. Statin efficacy did not vary with achieved CRP despite considerable variability in CRP response. The use of CRP as an indicator of efficacy of statin therapy on CVD risk in patients with type 2 diabetes is not supported by these data.

*Trial registration* NCT00327418

**Electronic supplementary material:**

The online version of this article (doi:10.1007/s00125-015-3586-8) contains peer-reviewed but unedited supplementary material, which is available to authorised users.

## Introduction

Statins have been shown to be beneficial in reducing cardiovascular disease (CVD) in patients with type 2 diabetes [1, 2], but it remains unclear whether these effects can be explained by their lipid-lowering effects only. Several studies show that statins reduce the inflammatory biomarker high-sensitivity C-reactive protein (CRP), an effect that is thought to be independent of change in LDL-cholesterol, based on low correlations (<0.1) between statin change in CRP and LDL-cholesterol [3–6].

Several studies have also suggested that part of the effect of statins on CVD is mediated through lowering of CRP concentrations [6–10]. JUPITER, The Justification for the Use of Statins in Primary Prevention: an Intervention Trial Evaluating Rosuvastatin, demonstrated, in 15,548 study participants with elevated CRP levels (≥2 mg/l), greater relative reductions in CVD in those achieving target post-treatment LDL-cholesterol and CRP levels, compared with an LDL-cholesterol target only, with rosuvastatin 20 mg/day [6]. These data have been used both as supportive evidence for developing specific CRP-reducing therapies for preventing CVD and also to suggest the clinical utility of CRP levels as targets for statin therapy alongside LDL-cholesterol levels. However, other statin trials, such as the Anglo-Scandinavian Cardiac Outcomes Trial (ASCOT) Lipid-Lowering Arm have not supported these findings [11, 12]. The most recent ASCOT analyses showed that CVD risk reductions with statin therapy did not vary by achieved CRP levels in hypertensive patients treated with atorvastatin for 6 months [12]. In the Heart Protection Study, baseline CRP levels did not predict statin response on CVD events in over 20,000 men and women treated with simvastatin or placebo during 5 years [13].

It is controversial whether CRP should be used as a target biomarker to assess the effects of statin treatment [14]. Most of these previous studies [6, 12, 13] primarily evaluated patients without type 2 diabetes. Baseline and not on-treatment CRP levels were analysed in the Heart Protection Study [13]; JUPITER included only people with initial CRP levels >2 mg/l and thus was unable to investigate lower CRP levels. As there is evidence that CRP levels are higher in patients with type 2 diabetes, we investigated whether there was evidence to support the clinical relevance of changing CRP levels during 1 year of follow-up with low-dose atorvastatin in a diabetes-specific trial without restrictions on the initial CRP level.

Specifically, we investigated whether CRP predicted CVD events, whether atorvastatin 10 mg daily lowered CRP and whether the effects of atorvastatin on CVD differed by achieved post-treatment CRP levels in a large sample of patients with type 2 diabetes from the Collaborative Atorvastatin Diabetes Study (CARDS). To directly compare results for CRP, all associations were also investigated for LDL-cholesterol.

## Methods

We performed a secondary analysis of CARDS, a multicentre, randomised, placebo-controlled trial that tested whether atorvastatin 10 mg daily reduced the incidence of cardiovascular outcomes in patients with type 2 diabetes [1, 15, 16]. Patients were randomised between November 1997 and June 2001. The study was conducted in accordance with the Declaration of Helsinki and the guidelines on good clinical practice. Each centre obtained local research ethics committee approval following approval from the Multicentre Research Ethics Committee. All patients gave fully informed written consent.

### Study population

Details of the trial have previously been published [1, 15, 16]. In brief, the total CARDS study population consisted of 2,838 type 2 diabetic men and women, aged 40–75 years, without prior history of myocardial infarction, unstable angina, coronary vascular surgery, cerebrovascular accident or severe peripheral vascular disease (defined as warranting surgery). All patients were required to have serum LDL-cholesterol ≤ 4.14 mmol/l (160 mg/dl) and serum triacylglycerol ≤ 6.78 mmol/l (600 mg/dl) at inclusion. Type 2 diabetes was defined using 1985 World Health Organization criteria [17].

### Laboratory measurements

Blood samples were collected after patients had fasted for at least 12 h. All biochemical and haematological measurements were made by a central laboratory at the Manchester Royal Infirmary, Manchester, UK. Of the total 2,838 participants, 374 were excluded due to lack of CRP data, leaving 2,464 participants for analysis. Of these 2,464 participants, 142 did not have a 1 year CRP measurement, leaving 2,322 participants with both baseline and 1 year CRP measurements. All baseline CRP and LDL-cholesterol samples were taken at randomisation before the study drug was given. Follow-up CRP measurements were all taken at the same time on stored samples using a defined visit schedule (366 days post randomisation), with the timing being allowed to vary within an agreed window of between 276 and 457 days post randomisation.

CRP concentrations were assayed in EDTA plasma samples by immunonephelometric methods using a BN II analyser (Siemens-Dade-Behring, Marburg, Germany). Laboratory reference values were 0–28.6 nmol/l (3 mg/l) for CRP. Intra- and interassay coefficients of variation were <8% for CRP. Lipaemic samples were diluted twofold with saline before centrifugation in 1 ml Eppendorf Tubes using an MSE Microfuge (London, UK) for 3 min at 14,000*g*. CRP was assayed in the infranatant.

Serum cholesterol and triacylglycerol concentrations were assessed by an automated enzymatic method. LDL-cholesterol was calculated using the Friedewald formula as described previously [1].

### Study outcomes

Fatal and non-fatal major CVD events included acute coronary heart disease (myocardial infarction, including silent infarction), unstable angina, acute coronary heart disease death, resuscitated cardiac arrest, coronary revascularisation procedures (coronary artery bypass graft surgery, percutaneous transluminal coronary angioplasty) or stroke as previously described [1, 15]. Any CVD event included major CVD, other cardiac death, hospitalised angina, non-fatal transient ischaemic attack, other non-fatal CVD or peripheral vascular disease events. An independent endpoint committee reviewed all cardiovascular events and deaths and classified them according to criteria specified in a detailed endpoint protocol. In addition to clinical events, annual ECGs were Minnesota-coded by two observers blinded to each other’s coding for the detection of silent myocardial infarction, and changes were confirmed as significant by a panel of cardiologists.

### Statistical analysis

To examine the effect of atorvastatin on CRP levels achieved at 1 year, we fitted linear regression models to the log-transformed CRP values 1 year post randomisation and included treatment group and log baseline CRP as explanatory variables; the percentage difference in CRP due to treatment was obtained from the β-coefficient for atorvastatin (by taking the exponential, subtracting 1 and multiplying by 100). To estimate the absolute treatment difference we fitted similar models using median regression. Two model adjustments were presented: model 1, for age and sex; and model 2, additionally for race (white vs non-white), smoking status (current vs none/past), systolic blood pressure, BMI, HbA_1c_, change in LDL-cholesterol, and days between randomisation and CRP measurement. Intention-to-treat analysis was compared with on-treatment analysis to check for bias due to participants not taking statins.

Pearson correlation coefficients were computed to test whether CRP levels were correlated with LDL-cholesterol levels using log-transformed CRP values, separately for baseline, 1 year and changes over time, and to test correlations with other cardiovascular risk factors.

To test the association between baseline CRP and incidence of (major/any) CVD we used Cox proportional hazards models. These models were adjusted for age, sex and treatment allocation in model 1, and additionally for race, smoking status, baseline systolic blood pressure, BMI, HbA_1c_ and LDL-cholesterol in model 2. We tested associations with incident CVD for achieved 1 year CRP and changes in CRP by modelling the time from 1 year CRP measurements to the first subsequent CVD event. Those with CVD events within the first year were excluded from analyses. Of 2,322 study participants, ten had missing LDL-cholesterol at 1 year, one had an implausible LDL-cholesterol measurement, one was lost to follow-up and 52 had CVD within the first year, leaving 2,258 participants for analysis. Model 2 extended with all confounders further dropped 35 participants from the analyses, leaving 2,223 participants for analysis. CRP was also examined as a continuous variable (per 1 SD increase in log_*e*_-transformed CRP) and by tertiles or quartiles for non-linearity. Similar multivariable Cox proportional hazards models were used to examine associations between baseline, 1 year and changes in LDL-cholesterol and incident (major/any) CVD.

To examine whether the effect of atorvastatin on major (or any) CVD varied by strata of achieved 1 year levels of CRP and LDL-cholesterol, and by combinations of achieved CRP and LDL-cholesterol, we used Cox proportional hazards models to estimate the HRs for CVD compared with placebo. For comparability with guidelines [18, 19] and earlier reports to define these strata, post-treatment CRP and LDL-cholesterol were dichotomised at various cut-points including post-treatment above and below median levels, and above and below specific target levels as in JUPITER [6] or ASCOT [11, 12]. Several adjustments were performed in model 0 for baseline CRP or LDL-cholesterol; in model 1 for age and sex; and, in model 2, further adjustment for race, smoking status, systolic blood pressure, BMI and HbA_1c_. We formally tested for interactions between the effect of atorvastatin and CRP or LDL-cholesterol by adding interaction terms (atorvastatin × CRP above/below targets) to the Cox models.

## Results

During 3.8 years of follow-up, 147 major CVD events and 244 any CVD events occurred in 2,322 participants with CRP data. The mean age of the participants was 61.6 years (SD 8.1) and 69% were male. A comparison of the baseline characteristics by treatment arm in those with CRP data (electronic supplementary material [ESM] Table 1) showed that both treatment arms were well balanced in terms of all characteristics, except for slightly lower baseline CRP levels in the atorvastatin compared with the placebo arm. In both arms combined, the median (interquartile range, IQR) baseline CRP levels were 13.3 (5.8, 31.9) nmol/l [1.4 (0.6, 3.4) mg/l]. The baseline median LDL-cholesterol concentration was 3.1 (2.6, 3.6) mmol/l [118.4 (99.8, 137.6) mg/dl]. Baseline CRP showed expected correlations with baseline risk CVD factors (ESM Table 2): for example, log_*e*_ CRP was positively associated with BMI (Pearson correlation coefficient 0.24, *p* < 0.001).

### Effects of atorvastatin on CRP

The atorvastatin arm showed a 9.8% reduction in CRP at 1 year (from baseline; median within-person change; Table 1) compared with an 18.5% increase in the placebo group. The net effect of atorvastatin on CRP levels was −32% (*p* < 0.0001) compared with placebo. By comparison, atorvastatin reduced LDL-cholesterol levels by 42% (*p* < 0.0001) from baseline to 1 year. Only four people in the atorvastatin group and seven in the placebo group were not on treatment 1 year after randomisation. Restricting the analyses to those still on treatment gave similar results to those produced in the intention-to-treat analysis.

**Table 1:** Effect of atorvastatin therapy on CRP concentrations in 2,322 patients with type 2 diabetes

Intention-to-treat analysis	Placebo (*n* = 1,148)	Atorvastatin (*n* = 1,174)
CRP, nmol/l [mg/l]		
Baseline, median (25th, 75th percentile)	14.5 (5.8, 33.8) [1.5 (0.6, 3.6)]	12.6 (5.9, 29.4) [1.3 (0.6, 3.1)]
12 months, median (25th, 75th percentile)	17.2 (8.0, 39.2) [1.8 (0.8, 4.1)]	11.9 (5.1, 27.3) [1.2 (0.5, 2.9)]
Change, % median (25th, 75th percentile)	18.5 (−41.4, 204.8)	−9.8 (−56.5, 115.2)
Net treatment effect		
ANCOVA using log-transformed CRP values, % difference in CRP compared with placebo (95% CI)		
Model 1^a^	−29.9 (−35.9, −23.3)^*^	
Model 2^b^	−32.0 (−40.4, −22.4)^*^	
ANCOVA using median regression of untransformed CRP values, absolute difference in CRP compared with placebo (95% CI)		
Model 1^a^, nmol/l [mg/l]	−4.9 (−6.1, −3.8) [−0.5 (−0.6, −0.4)]^*^	
Model 2^b^, nmol/l [mg/l]	−4.4 (−6.4, −2.3) [−0.5 (−0.7, −0.2)]^*^	

^a^Model 1, age and sex

^b^Model 2, race, smoking status, systolic blood pressure, BMI, HbA_1c_, change in LDL-cholesterol, days between randomisation and CRP measurement; this model is based on 2,311 participants with complete covariate data

*p < 0.0001 vs placebo

Although there was a net treatment effect on CRP, baseline CRP and changes from baseline were much more variable than LDL-cholesterol. The median (IQR) within-person absolute change in CRP on statin was −1.0 (−9.4, 6.9) nmol/l [−0.11 (−0.99, 0.72) mg/l], with 45% of those on statin having no fall in CRP (median [IQR] within-person per cent change −9.8% [−56, +115]. By contrast, the LDL-cholesterol response was much less variable, with a median (IQR) fall in LDL-cholesterol in 96% of people on the statin of 41% (−51%, −31%). Furthermore, the correlation coefficients between baseline log-transformed CRP and LDL-cholesterol levels were low at 0.04 (*p* = 0.03). Low, although statistically significant, correlations were also found between on-treatment CRP and LDL-cholesterol and change in log-transformed CRP and LDL-cholesterol (0.14 [*p* < 0.0001] and 0.07 [*p* = 0.001], respectively).

### CRP, LDL-cholesterol and CVD

We analysed the relationship between CRP and CVD risk in all patients, using a linear term for CRP (per 1 SD in log_*e*_ CRP). No relationship was seen for baseline, 1 year or change in CRP over time (HR ∼1 for major or any CVD). Stratified by treatment group, there was a weak, non-significant association (HR 1.17 [95% CI 0.94, 1.45]) between baseline CRP (per 1 SD in log_*e*_ CRP) and incidence of any CVD event in the atorvastatin group only, whereas in the placebo group the HR was 0.95 (95% CI 0.80, 1.13) after adjustment for other covariates beyond age and sex. In case there was a non-linear relationship we also categorised baseline CRP levels into tertiles (Table 2): no significant relationship between CRP and CVD risk adjusted for treatment was found, except for the composite event of any CVD (HR_top vs bottom tertile_ 1.35 [95% CI 0.98, 1.86]), and this apparent effect was no longer significant after adjustment for other covariates beyond age and sex. Baseline LDL-cholesterol levels predicted major CVD independently of age, sex, treatment, race, smoking status, systolic blood pressure, BMI and HbA_1c_, with an HR of 1.21 (95% CI 1.02, 1.44) (Table 2). Similar associations were found with any CVD, for baseline, 1 year LDL-cholesterol levels and changes in LDL-cholesterol levels.

**Table 2:** Event rates across tertiles of baseline CRP and LDL-cholesterol in the CARDS population

Event type by tertile or per unit SD	Events/persons (event rate/100 person-years)	Model 1^a^		Model 2^b^	
		HR (95% CI)	*p* value	HR (95% CI)	*p* value^d^
CRP^c^					
Major cardiovascular event (*n* = 147)					
Tertile 1, <7.9 nmol/l	52/772 (1.72)	1		1	
Tertile 2, 7.9–23.8 nmol/l^e^	55/776 (1.89)	1.19 (0.82, 1.75)		1.11 (0.75, 1.63)	
Tertile 3, ≥23.8 nmol/l	40/774 (1.38)	1.05 (0.69, 1.60)	0.755	0.88 (0.57, 1.35)	0.589
SD unit of log CRP		0.97 (0.82, 1.15)	0.730	0.89 (0.75, 1.06)	0.205
Any cardiovascular event (*n* = 244)					
Tertile 1, <7.9 nmol/l	76/772 (2.55)	1		1	
Tertile 2, 7.9–23.8 nmol/l^e^	87/776 (3.07)	1.27 (0.93, 1.73)		1.17 (0.86, 1.60)	
Tertile 3, ≥23.8 nmol/l	81/774 (2.87)	1.35 (0.98, 1.86)	0.061	1.14 (0.82, 1.58)	0.439
SD unit of log CRP		1.10 (0.97,1.25)	0.151	1.02 (0.89, 1.17)	0.749
LDL-cholesterol^c^					
Major cardiovascular event (*n* = 147)					
Tertile 1, <2.75 mmol/l	44/773 (1.49)	1		1	
Tertile 2, 2.75–3.40 mmol/l	45/774 (1.54)	1.07 (0.71, 1.62)		1.03 (0.68, 1.56)	
Tertile 3, ≥3.40 mmol/l	58/774 (1.97)	1.44 (0.97, 2.13)	0.066	1.44 (0.97, 2.14)	0.065
SD unit of LDL-cholesterol		1.21 (1.02, 1.43)	0.029	1.21 (1.02, 1.44)	0.027
Any cardiovascular event (*n* = 244)					
Tertile 1, <2.75 mmol/l	73/773 (2.53)	1		1	
Tertile 2, 2.75–3.40 mmol/l	78/774 (2.73)	1.10 (0.80, 1.51)		1.06 (0.77, 1.46)	
Tertile 3, ≥3.40 mmol/l	93/774 (3.22)	1.33 (0.98, 1.82)	0.064	1.35 (0.99, 1.84)	0.054
SD unit of LDL-cholesterol		1.15 (1.01, 1.31)	0.036	1.16 (1.02, 1.32)	0.026

^a^Model 1, age, sex and treatment allocation

^b^Model 2, model 1 + race, smoking status, systolic blood pressure, BMI, HbA_1c_ and LDL-cholesterol for association between CRP and (major/any) CVD

^c^ *n* = 2,322 for CRP analyses and *n* = 2,321 for LDL-cholesterol analyses

^d^ *p* values are from likelihood ratio tests

^e^7.9–23.8 nmol/l = 0.83–2.49 mg/l

### Effect of atorvastatin on CVD by achieved lowering of CRP or LDL-cholesterol

The median (IQR) achieved LDL-cholesterol level was 3.12 (2.58, 3.64) mmol/l [121 (100, 141) mg/dl] in the placebo arm and 1.79 (1.41, 2.23) mmol/l [60 (65, 86) mg/dl] in the atorvastatin arm. The median achieved CRP level was 17.2 (8.0, 39.1) nmol/l [1.81 (0.84, 4.12) mg/l] in the placebo arm and 11.8 (5.0, 27.2) nmol/l [1.24 (0.53, 2.86) mg/l] in the atorvastatin arm. Compared with the placebo group, the reduction in CVD with atorvastatin was not significantly different by categories of 1 year LDL-cholesterol or CRP levels achieved at any binary cut-point used (Table 3). The HRs of the effect of atorvastatin vs placebo on CVD risk were 0.5–0.7, which is consistent with the overall effect in the total trial population, including those without CRP data [1]. There was no evidence of significant interactions (all *p* ≥ 0.9) between the overall efficacy of atorvastatin on major CVD events and achieved CRP above/below targets or LDL-cholesterol above/below targets. These results persisted after adjustment for age, sex, smoking status, race, systolic blood pressure, BMI and HbA_1c_. Nor did we find any significant differences in effects on incidence of any CVD by category of achieved LDL-cholesterol or CRP.

**Table 3:** Effect of atorvastatin on CVD events by level of CRP or LDL-cholesterol achieved at 1 year

CVD event (*N* = 2,258)	Events/persons (event rate/100 person-years)	Model 0^a^, adjusted HR (95% CI)	Model 1^b^, adjusted HR (95% CI)	Model 2^c^, adjusted HR (95% CI)
Major cardiovascular event (116 cases)				
Placebo (74 cases)	74/1,115 (2.33)	1	1	1
Atorvastatin (42 cases)				
LDL-c <2 mmol/l [77 mg/dl]	28/719 (1.32)	0.54 (0.35, 0.83)	0.58 (0.37, 0.89)	0.58 (0.37, 0.90)
LDL-c ≥2 mmol/l [77 mg/dl]	14/424 (1.18)	0.58 (0.33, 1.03)	0.51 (0.28, 0.92)	0.51 (0.28, 0.91)
CRP <19.0 nmol/l [2 mg/l]	30/752 (1.35)	0.55 (0.36, 0.83)	0.53 (0.35, 0.82)	0.55 (0.36, 0.84)
CRP ≥19.0 nmol/l [2 mg/l]	12/391 (1.12)	0.57 (0.31, 1.06)	0.59 (0.32, 1.09)	0.54 (0.29, 1.01)
CRP fell by year 1	22/624 (1.22)	0.53 (0.33, 0.85)	0.54 (0.33, 0.86)	0.54 (0.34, 0.88)
CRP did not fall	20/519 (1.34)	0.58 (0.35, 0.95)	0.56 (0.34, 0.93)	0.55 (0.33, 0.91)
LDL-c <1.8 mmol/l [70 mg/dl]	19/577 (1.10)	0.45 (0.27, 0.74)	0.48 (0.29, 0.80)	0.48 (0.29, 0.81)
LDL-c ≥1.8 mmol/l [70 mg/dl]	23/566 (1.46)	0.69 (0.43, 1.10)	0.63 (0.39, 1.03)	0.63 (0.39, 1.02)
CRP <9.5 nmol/l [1 mg/l]	20/500 (1.30)	0.50 (0.30, 0.82)	0.49 (0.30, 0.80)	0.51 (0.31, 0.84)
CRP ≥9.5 nmol/l [1 mg/l]	22/643 (1.24)	0.61 (0.38, 0.99)	0.62 (0.38, 1.00)	0.58 (0.36, 0.94)
% Change in LDL-c < median^d^	21/588 (1.19)	0.50 (0.31, 0.81)	0.50 (0.30, 0.80)	0.50 (0.31, 0.82)
% Change in LDL-c ≥ median	21/555 (1.37)	0.62 (0.38, 1.01)	0.62 (0.38, 1.01)	0.61 (0.37, 1.00)
% Change in CRP < median^e^	20/571 (1.20)	0.51 (0.31, 0.84)	0.52 (0.32, 0.85)	0.52 (0.32, 0.86)
% Change in CRP ≥ median	22/572 (1.35)	0.60 (0.37, 0.96)	0.58 (0.36, 0.94)	0.57 (0.35, 0.92)
Any cardiovascular event (190 cases)				
Placebo (116 cases)	116/1,115 (3.73)		1	1
Atorvastatin (74 cases)				
LDL-c <2 mmol/l [77 mg/dl]	44/719 (2.10)	0.55 (0.39, 0.78)	0.58 (0.41, 0.83)	0.59 (0.41, 0.84)
LDL-c ≥2 mmol/l [77 mg/dl]	30/424 (2.59)	0.76 (0.50, 1.14)	0.67 (0.44, 1.03)	0.67 (0.44, 1.02)
CRP <19.0 nmol/l [2 mg/l]	52/752 (2.37)	0.61 (0.44, 0.85)	0.62 (0.45, 0.86)	0.64 (0.46, 0.89)
CRP ≥19.0 nmol/l [2 mg/l]	22/391 (2.08)	0.62 (0.39, 0.99)	0.62 (0.39, 0.99)	0.58 (0.36, 0.93)
CRP fell by year 1	44/624 (2.49)	0.68 (0.48, 0.96)	0.67 (0.48, 0.96)	0.67 (0.47, 0.95)
CRP did not fall	30/519 (2.03)	0.54 (0.36, 0.82)	0.55 (0.37, 0.83)	0.56 (0.37, 0.84)
LDL-c <1.8 mmol/l [70 mg/dl]	31/577 (1.81)	0.47 (0.32, 0.70)	0.50 (0.33, 0.75)	0.51 (0.34, 0.77)
LDL-c ≥1.8 mmol/l [70 mg/dl]	43/566 (2.80)	0.80 (0.56, 1.14)	0.74 (0.51, 1.06)	0.73 (0.51, 1.06)
CRP <9.5 nmol/l [1 mg/l]	37/500 (2.46)	0.61 (0.42, 0.89)	0.62 (0.43, 0.90)	0.65 (0.45, 0.95)
CRP ≥9.5 nmol/l [1 mg/l]	37/643 (2.12)	0.62 (0.43, 0.90)	0.62 (0.43, 0.90)	0.59 (0.40, 0.86)
% Change in LDL-c < median^d^	38/588 (2.19)	0.58 (0.40, 0.84)	0.57 (0.40, 0.83)	0.59 (0.41, 0.85)
% Change in LDL-c ≥ median	36/555 (2.38)	0.66 (0.45, 0.97)	0.67 (0.46, 0.97)	0.65 (0.45, 0.96)
% Change in CRP < median^e^	42/571 (2.58)	0.69 (0.48, 0.98)	0.69 (0.48, 0.98)	0.68 (0.48, 0.97)
% Change in CRP ≥ median	32/572 (1.98)	0.54 (0.36, 0.80)	0.55 (0.37, 0.82)	0.53 (0.37, 0.83)

^a^Model 0, age and sex

^b^Model 1, model 0 + baseline LDL-cholesterol or CRP

^c^Model 2, model 1 + race, smoking status, systolic blood pressure, BMI and HbA_1c_

^d^The median (IQR) achieved LDL-cholesterol level was 3.12 (2.58, 3.64) mmol/l [121 (100, 141) mg/dl] in the placebo arm and 1.79 (1.41, 2.23) mmol/l [60 (65, 86) mg/dl] in the atorvastatin arm

^e^The median (IQR) achieved CRP level was 17.2 (8.0, 39.1) nmol/l [1.81 (0.84, 4.12) mg/l] in the placebo arm and 11.8 (5.0, 27.2) nmol/l [1.24 (0.53, 2.86) mg/l] in the atorvastatin arm

LDL-c, LDL-cholesterol

### Effect of atorvastatin on CVD by combinations of achieved CRP and LDL-cholesterol

Using a number of models, there was no clear evidence that the achievement of target CRP levels after the LDL-cholesterol target was achieved resulted in further reductions in risk (Table 4). Among patients with achieved LDL-cholesterol <1.8 mmol/l, atorvastatin reduced the risk of CVD events, irrespective of whether achieved CRP was ≥9.5 nmol/l or <9.5 nmol/l (1 mg/l). Similar HRs with overlapping CIs were found with 1 year achieved levels of LDL-cholesterol and CRP regardless of cut-points used. In summary, with overlapping CIs between all comparisons and non-significant *p* values for interaction (all above 0.6), there was no difference in the effect of atorvastatin vs placebo on CVD events in any of the combined groups of achieved 1 year LDL-cholesterol or CRP targets.

**Table 4:** Effect of atorvastatin on CVD categorised by combined 1 year achievement of LDL-cholesterol and CRP levels

CVD event (*N* = 2,258)	Events/persons	Event rate (per 100 person-years)	Adjusted HR^a^ (95% CI)
Major cardiovascular event			
Placebo	74/1,115	2.33	1
Atorvastatin subgroups			
LDL-c <1.8 mmol/l, CRP <19.0 nmol/l	14/400	1.14	0.44 (0.25, 0.78)
LDL-c <1.8 mmol/l, CRP ≥19.0 nmol/l	16/352	1.59	0.69 (0.40, 1.19)
LDL-c ≥1.8 mmol/l, CRP <19.0 nmol/l	5/177	0.98	0.47 (0.19, 1.16)
LDL-c ≥1.8 mmol/l, CRP ≥19.0 nmol/l	7/214	1.24	0.68 (0.31, 1.47)
Any cardiovascular event			
Placebo	116/1,115	3.73	1
Atorvastatin subgroups			
LDL-c <1.8 mmol/l, CRP <19.0 nmol/l	24/400	1.99	0.55 (0.35, 0.86)
LDL-c <1.8 mmol/l, CRP ≥19.0 nmol/l	28/352	2.84	0.73 (0.48, 1.12)
LDL-c ≥1.8 mmol/l, CRP <19.0 nmol/l	7/177	1.38	0.41 (0.19, 0.89)
LDL-c ≥1.8 mmol/l, CRP ≥19.0 nmol/l	15/214	2.72	0.69 (0.39, 1.23)

^a^Adjusted for age, sex, baseline LDL-cholesterol, baseline CRP, race, smoking status, systolic blood pressure, BMI and HbA_1c_

LDL-c, LDL-cholesterol

## Discussion

In this study we found that CRP was not an important predictor of CVD in patients with type 2 diabetes. There was a net lowering of CRP levels by atorvastatin 10 mg daily in this primary prevention diabetic population, with 32% lower CRP levels at 1 year. However, there was considerably greater variability in CRP than in LDL-cholesterol response. The magnitude of statin efficacy in the prevention of CVD did not differ by achieved on-treatment CRP levels. Combining LDL-cholesterol and CRP on-treatment categories demonstrated similar reduced relative risks of CVD by atorvastatin compared with placebo in all comparisons. Thus, CARDS does not yield any evidence to support the use of CRP as an indicator of efficacy of statin therapy on CVD risk in patients with type 2 diabetes.

We did not find any consistent association between baseline or post-treatment CRP levels and major (or any) CVD. Several prospective cohort studies in patients with type 2 diabetes [20–23] have demonstrated elevated CVD risk with higher CRP levels, with HRs ranging between 1.4 and 2.6. In previous studies, higher CRP levels were indicated on a continuous scale per SD log unit [23] or were divided into categories greater than 3 mg/l [21, 22] or by quartiles [20]. In most of these studies, however, CRP levels were much higher (ranging from 1.8 to 3 mg/l) than in our study population (median 1.4 mg/l). Our study population of patients with type 2 diabetes without prior CVD had intermediate CRP values and were at modest risk of CVD events [24]. Our findings are consistent with some studies that directly compared patients with diabetes with those without and found that associations between CRP and CVD were generally weaker and not significant in patients with type 2 diabetes compared with non-diabetic individuals [25–27]. Their explanation was that other CVD risk factors typical for patients with type 2 diabetes, such as high triacylglycerol levels, low HDL-cholesterol levels, hypertension and hyperglycaemia, partially mask the role of CRP as a risk factor for CVD, which could also contribute to the lack of association with CVD in our study. In contrast with the results for CRP, both baseline and on-treatment LDL-cholesterol were clear predictors of CVD.

We did find a net lowering of CRP levels with atorvastatin 10 mg daily in patients with type 2 diabetes. Primary prevention trials with various statins and varying study periods in non-diabetic patients previously demonstrated that statin therapy significantly decreased levels of CRP and showed significant net treatment effects compared with placebo, ranging between −27% and −37%, which was comparable with our treatment effect of −32% [6, 11, 13]. We also found a low correlation between CRP change and LDL-cholesterol change with statin. This low correlation has been asserted in previous studies to be consistent with the pleiotropic effects of statins. However, it may also simply reflect that CRP levels are much more variable than LDL-cholesterol and that change in CRP with statin is much more variable than change in LDL-cholesterol. Consistent with this, we found a wide variability in the change in CRP on statin, with almost half (45%) of those on statin not having any fall in CRP and a narrow variability in LDL-cholesterol, as almost all participants (96%) had a fall in LDL-cholesterol by atorvastatin with a narrow IQR in response. In order to demonstrate whether changes in LDL-cholesterol or CRP associated with treatment relate to the magnitude of the treatment effect of atorvastatin on CVD there needs to be considerable variation among those assigned to atorvastatin in the change observed in that biomarker. Thus, the power to demonstrate variability in the HR for CVD with atorvastatin is considerably less here for LDL-cholesterol than for CRP. Despite this, we could not find any variability in the CVD response by either achieved CRP or LDL-cholesterol.

A meta-regression of 23 placebo-controlled trials previously noted the lack of support for a pleiotropic effect and demonstrated that at least 90% of the CRP reduction detected with therapies to lower LDL-cholesterol can be explained by reductions in LDL-cholesterol [28].

We found no clear evidence that the efficacy of atorvastatin on CVD varied by achieved CRP level. This lack of association has been reported previously in ASCOT [11, 12]. ASCOT investigators demonstrated both in a nested case–control design [11] and a more recent cohort design [12] that in hypertensive patients neither baseline nor achieved CRP levels with atorvastatin 10 mg/day predicted the efficacy of statin treatment in preventing future CVD. On the contrary, JUPITER [6] (*n* = 15,548) demonstrated that rosuvastatin 20 mg daily reduced CVD by 55% during a median follow-up period of 1.9 years in those who achieved on-treatment LDL-cholesterol levels <1.8 mmol/l, by 62% in those achieving CRP below 2 mg/l, and by 65% in those achieving both LDL-cholesterol and CRP below target levels, supporting the non-lipid benefits of statins. Patients with type 2 diabetes and those with lower CRP levels were not investigated. However, levels of CRP at baseline in CARDS were less than half those in JUPITER [6], with a baseline median CRP of 4.3 mg/l in the placebo group, compared with 1.4 mg/l in our study, as JUPITER selected men and women with a high baseline CRP level (>2 mg/l). This may in part explain the discrepancy in findings between our study and those of JUPITER.

Strengths of our study are the large number of patients with type 2 diabetes and a relatively long follow-up time (almost 4 years), with no constraints on CRP levels at baseline. The study findings can be generalised to a wider population of patients with type 2 diabetes. Although the inclusion criteria required that for individuals to be eligible for randomisation one or more additional risk factors for CVD should be present, we think that in fact most patients with type 2 diabetes probably have at least one risk factor anyway [1]. Limitations are the relatively few CVD events due to early termination of CARDS because of the emergence of clear evidence of benefit. Therefore, major and any CVD (including peripheral vascular disease) were included as study outcomes. Due to limited events, subgroup analyses of the effect of statin therapy on CVD events by combined achieved LDL-cholesterol and CRP levels must be interpreted carefully.

In summary, we did not find any evidence to support the idea that reduction of CVD by statins in patients with modest CRP levels is mediated by lowering CRP. The use of CRP as an indicator of the efficacy of statin therapy on CVD risk in patients with type 2 diabetes is not supported by these data.

Two large placebo-controlled trials using targeted anti-inflammatory drugs for secondary prevention of myocardial infarction have been initiated, namely the Canakinumab Anti-inflammatory Thrombosis Outcomes Study (CANTOS) and the Cardiovascular Inflammation Reduction Trial (CIRT) [29]. Fundamental evidence for the CVD benefits of reducing inflammation must await these trials.

## Electronic supplementary material

ESM List of CARDS Investigators (PDF 75.2 kb)

ESM Table 1 (PDF 23.3 kb)

ESM Table 2 (PDF 15.5 kb)

## Abbreviations

ASCOT: Anglo-Scandinavian Cardiac Outcomes Trial
CARDS: Collaborative Atorvastatin Diabetes Study
CRP: C-reactive protein
CVD: Cardiovascular disease
IQR: Interquartile range
JUPITER: Justification for the Use of Statins in Primary Prevention: an Intervention Trial Evaluating Rosuvastatin
