# Supplementary material for: Effect of atorvastatin on C-reactive protein and benefits for cardiovascular disease in patients with type 2 diabetes: analyses from the Collaborative Atorvastatin Diabetes Trial
Source: Diabetologia. 2015 Apr 22;58(7):1494–502. doi: 10.1007/s00125-015-3586-8 (PMC4472939; doi:10.1007/s00125-015-3586-8)
Supplement: Supplementary file 1 — (PDF 75.2 kb) [file 125_2015_3586_MOESM1_ESM.pdf]

ESM List of CARDS Investigators

CARDS committee members
Steering Committee—B Pentecost (chairman), Birmingham; J Betteridge (principal investigator) London; H Colhoun (principal investigator) Dublin; P Durrington (principal investigator) Manchester; J Fuller (principal investigator) London; A Gotto, New York; G Hitman (principal investigator) London; D Julian, London; D Lambert, Department of Health, Leeds; K Lloyd, Pfizer UK, Tadworth; M Murphy, Diabetes UK, London; A Neil (principal investigator) Oxford; C Newman, Pfizer USA, New York; K Pyörälä, Kuopio.

Endpoint Committee—J Jarrett (chairman) London; S Hardman, London; M Marber, London.

Safety Committee—H Keen (chairman) London; P Clifton, Teignmouth; M Laker, Newcastle upon Tyne; S Senn, Glasgow.

Key staff at central laboratory
P Durrington, M France, M Mackness, V Menys, A Moorhouse, R Pope, H Prais, J Seneviratne.
Electrocardiography coding
B Peachey, London; S Taylor, St Albans.
Key staff at Pfizer UK
G Lewis, I Martin.
Key staff at UCL coordinating centre
H Colhoun, W Dodds, R Fox, J Fuller, S Livingstone, B Starr, M Thomason, D Webb, A West.
Clinical centres and investigators
Aberdeen Royal: J Broom; Studholme Medical Centre, Ashford: S Butt, K Tang; The Surgery, Ayr: B Lennox; Ayr Hospital: A Collier; Beehive Surgery, Bath: J Hampton; Oldfield Surgery, Bath: T J Harris, GD Walker; Pulteney Street Surgery, Bath: P J Tilley; Royal United, Bath: J Reckless; St Chad's Surgery, Bath: E J Widdowson; St James' Surgery, Bath: I M Orpen; Belfast City: M S Fetherston, J R Hayes; Royal Victoria, Belfast: D R McCance; Medical Centre, Chelmsley Wood, Birmingham: D M Allin; Birmingham Heartlands: P Dodson; Queen Elizabeth, Birmingham: U Martin; Synexus Limited, Birmingham: G S Jassel, M Salman; Bolton Diabetes Centre: J Dean; Bottreaux Practice, Boscastle: G D Garrod, C Jarvis; Royal Bournemouth: S Egan, D Kerr; St Alban's Medical Centre, Bournemouth: I Nelemans; Health Centre, Bradford on Avon: J S Heffer; Frenchay, Bristol: C J Burns-Cox, V J Parfitt; Addenbrookes, Cambridge: M J Brown; Synexus Limited, Cardiff: C Godfrey, G L Newcombe; St Helier, Carshalton: J Barron; Aspire Research Limited, Chesterfield: M Blagden; Rowden Surgery, Chippenham: R M C Gaunt; Porch Surgery, Corsham: A Cowie; Coventry & Warwickshire: E Hillhouse; Bridge Medical Centre, Crawley: A L Cooper; Pound Hill Surgery, Crawley: N W Jackson; Derby City: R. Donnelly, A R Scott; Dewsbury District: T Kemp, C Rajeswaren; St James', Dublin: J Nolan; Dumfries & Galloway Royal: J R Lawrence; St Michael's, Dun Laoghaire: M J McKenna; Muirhead Medical Centre, Dundee: B Kilgallon; Ninewells, Dundee: G P Leese, A D Morris; Hairmyres, East Kilbride: S J Benbow, H Cohen, D Mathews; Edinburgh Royal: V McAuley, J D Walker; Western General, Edinburgh: J A McKnight; St Margaret's, Epping: G B Ambepitiya; Epsom District: C Speirs; Health

Centre, Falmouth: A Rotheray, A Seaman, V L Wight; River Practice, Fowey: A Middleton; Frome Medical Practice: T E Cahill; Queen Elizabeth, Gateshead: A Syed, J Weaver; Medway Maritime, Gillingham: I Scobie; Gartnaval General, Glasgow: M Small; Glasgow Royal: J Gray, K R Paterson; Southern General, Glasgow: L Fraser, S J Gallacher; Victoria Infirmary, Glasgow: C M Kesson; Harrogate District: P Hammond; Hartlepool General: G Hawthorne, J MacLeod; St Thomas Surgery, Haverfordwest: R W G Thompson; Withybush General, Haverfordwest: N Jowett; Princess Royal, Haywards Heath: T Wheatley; Hemel Hempstead General: C Johnston; Hetton le Hole Medical Centre: M Baldasera, P A Dobson; Hildenborough Medical Group: P Goozee; Raigmore, Inverness: S MacRury; Townhead Surgery, Irvine: M F Doig, D D McKeith; Leicester General: A C Burden, R Gregory; Synexus Limited, Liverpool: J Robinson; Royal Liverpool & Broadgreen: J P Vora; St John’s at Howden, Livingstone: R S Gray; Charing Cross, London: C Leroux, M Seed; Hammersmith, London: A Dornhorst; North Middlesex, London: H Tindall; Royal Free, London: M Press; Symons Medical Centre, Maidenhead: R C F Symons; Hope, Manchester: R Young; North Manchester Diabetes Centre: P Wiles; Synexus Limited, Manchester: D Dev, J James; Trafford General, Manchester: WP Stephens; Giffords Primary Care Centre, Melksham: C H Lennon; Newcastle General: S Marshall, M W Stewart; Friarage, Northallerton: R Fisken, A Waise; Queens Medical Centre, Nottingham: P I Mansell, S Page; George Eliot, Nuneaton: V Patel; Southport & Ormskirk, Ormskirk: J. Horsley, R S Oelbaum; Royal Oldham: D. Bhatnagar; Churchill, Oxford: D Matthews, R. Spivey; Royal Alexandra, Paisley: B M Fisher, J Hinnie; Alverton Practice, Penzance: J F Ryan; Cape Cornwall Surgery, St. Just, Penzance: A Ellery, W Jago; Knowle House Surgery, Plymouth: K Gillespie, T Hall; Woolwell Medical Centre, Plymouth: C P Fletcher; Pontefract General: J Howell, C White; Royal Glamorgan, Pontypridd: M D Page; Queen Alexandra, Portsmouth: K M Shaw; Synexus Limited, Reading: M Horne, M Thomson; St Cross, Rugby: J P O’Hare; The Surgery, Ryde: E J Hughes; Brannel Surgery, St Austell: J R Cecil; Salisbury District: P Mansell, N O’Connell; Saltash Health Centre: R C Cook; Scunthorpe General: S Beer; Carterknowle & Dore Medical Practice, Sheffield: B King; Norwood Medical Centre, Sheffield: P Hardy; Southey Green Medical Centre, Sheffield: N H Patel; Royal Shrewsbury: A MacLeod; Chiltern International Limited, Slough: M MacMahon, P Palmer; Brook Lane Surgery, Southampton: T M Tayler; Royal South Hants, Southampton: B Leatherdale; Queensway Surgery, Southend on Sea: D Sills; Lister, Stevenage: L J Borthwick; Royal, Stirling: C J G Kelly, S B M Reith; Huthwaite Medical Centre, Sutton-in-Ashfield: P Smith, E Ulliott; John Pease Diabetes Centre, Sutton-in-Ashfield: R Lloyd-Mostyn, K Sands; Cwmfelin Medical Centre, Swansea: P J Davies; St Helens Medical Centre, Swansea: P Cummings; Talybont Surgery, Swansea: P A Edwards, R M Ferry (deceased), A H Jones; Jubilee Surgery, Titchfield: PWG Evans; Bradford Road Medical Centre, Trowbridge: S C W Rowlands; Lovemead Group Practice, Trowbridge: M J B Duckworth; Treliske, Truro: S Fleming; Grosvenor Medical Centre, Tunbridge Wells: G J Charlwood; Pinderfields General, Wakefield: WBurr, D Nagi; The Avenue Surgery, Warminster: C H Browne, K R Bullen; Watford General: M Clements; Sandwell District General, West Bromwich: D A Robertson; Eastleigh Surgery, Westbury: R Edwards; Weston-super-Mare General: C Dayan; Queen Elizabeth II, Welwyn Garden City: P Winocour; Synexus Limited, Wigan: J Fraser; Wishaw General: I A D O’Brien; New Cross, Wolverhampton: B M Singh; Nottinghamshire Research Associates, Worksop: J A Fulton, L Millar, S Warner; Maelor, Wrexham: J N Harvey; York District: P Jennings, J Thow.
