# Supplementary material for: Effect of atorvastatin on C-reactive protein and benefits for cardiovascular disease in patients with type 2 diabetes: analyses from the Collaborative Atorvastatin Diabetes Trial
Source: Diabetologia. 2015 Apr 22;58(7):1494–502. doi: 10.1007/s00125-015-3586-8 (PMC4472939; doi:10.1007/s00125-015-3586-8)
Supplement: Supplementary file 2 — (PDF 23.3 kb) [file 125_2015_3586_MOESM2_ESM.pdf]

ESM Table 1 Baseline characteristics of 2,322 CARDS patients with type 2 diabetes by treatment arm

Variables (n=2,322)	Placebo (n=1,148)	Atorvastatin (n=1,174)	Adjusted P value
Baseline CRP (nmol/l) [mg/l]	14.5 (5.8, 33.8) [1.5 (0.6, 3.6)]	12.6 (5.9, 29.4) [1.3 (0.6, 3.1)]	0.042
Male	69.5 (798)	67.8 (796)	0.387
Age (years)	61.8 (7.9)	61.4 (8.3)	0.233
Duration of diabetes (years)	7.7 (6.3)	8.0 (6.3)	0.191
HbA1c (% / mmol/mol)	7.8 (1.4) / 62 (15)	7,9 (1.4) /62 (15)	0.341
BMI (kg/m^2)	28.9 (3.5)	28.7 (3.6)	0.087
UKPDS risk score	20 (12, 29)	20 (12, 30)	0.142
Framingham risk score	20 (13, 31)	20 (13, 31)	0.301
Systolic blood pressure (mm Hg)	144 (16)	144 (16)	0.851
Diastolic blood pressure (mm Hg)	83 (8)	83 (8)	0.245
Risk Factors (%)			
Current smoker	22.8 (261)	21.9 (257)	0.494
Hypertension	83.9 (963)	84.1 (987)	0.764
Microalbuminuria	14.3 (225)	21.0 (21)	0.418
Macroalbuminuria	1.7 (27)	6.0 (6)	0.344
Retinopathy	29.4 (337)	30.4 (357)	0.501
Lipids			
LDL cholesterol (mmol/l) [mg/dl]	3.02 (0.69) [117 (27)]	3.04 (0.72) [118 (28)]	0.404
Total cholesterol (mmol/l) [mg/dl]	5.34 (0.80) [206 (31)]	5.36 (0.83) [207 (32)]	0.654
Triacylglycerol (mmol/l) [mg/dl]	1.67 (1.17,2.43) [148 (103, 216)]	1.70 (1.20, 2.40) [151 (106, 213)]	0.853
HDL cholesterol (mmol/l) [mg/dl]	1.41 (0.34) [54 (13)]	1.39 (0.32) [54 (12)]	0.144
Apolipoprotein A-1 (g/l)	1.53 (0.29)	1.53 (0.27)	0.889
Apolipoprotein B (g/l)	1.15 (0.24)	1.17 (0.24)	0.240
Non-HDL cholesterol (mmol/l)[mg/dl]	3.93 (0.81) [152 (31)]	3.97 (0.82) [153 (32)]	0.307

Total Chol/HDL ratio	4.0 (1.0)	4.0 (1.0)	0.223
Medication			
Insulin or oral hypoglycaemic drugs	84.5 (970)	85.5 (1004)	0.546
Blood pressure lowering drugs	66.9 (768)	67.3 (790)	0.777
Aspirin or other antiplatelet drugs	14.4 (165)	16.0 (188)	0.226
Oral contraceptives or HRT	6.7 (77)	6.1 (72)	0.245
LDL-c at year 1 (mmol/l) [mg/dl]	3.08 (0.78)	1.83 (0.65)	<0.001
	[119.2 (30.3)]	[70.9 (25.1)]	
CRP at year 1 (nmol/l) [mg/l]	17.2 (8.0, 39.1)	11.8 (5.0, 27.2)	
	[1.8 (0.8, 4.1)]	[1.2 (0.5, 2.9)]	0.055

Values are % (n) for categorical variables, mean (SD) or median (interquartile range: 25th, 75th percentiles) where skewed for continuous variables. Wald test P values are from logistic regression models of treatment arm adjusted for age and sex. Hypertension: systolic/diastolic blood pressure >=140/90 mmHg or on blood pressure lowering drugs. P values for micro/macroalbuminuria with normoalbuminuria as the reference level. CRP, C-reactive protein. HbA1c, glycated haemoglobin. BMI, body mass index. UKPDS, United Kingdom Prospective Diabetes Study. LDL, low-density lipoprotein. HDL, high-density lipoprotein. Chol, cholesterol. HRT, hormone replacement therapy.
