# Supplementary material for: Effect of atorvastatin on C-reactive protein and benefits for cardiovascular disease in patients with type 2 diabetes: analyses from the Collaborative Atorvastatin Diabetes Trial
Source: Diabetologia. 2015 Apr 22;58(7):1494–502. doi: 10.1007/s00125-015-3586-8 (PMC4472939; doi:10.1007/s00125-015-3586-8)
Supplement: Supplementary file 3 — (PDF 15.5 kb) [file 125_2015_3586_MOESM3_ESM.pdf]

ESM Table 2 Pearson correlation of log transformed baseline CRP with baseline characteristics of 2,322 CARDS patients with type 2 diabetes

Variables (n=2,322)	Pearson correlation with $\log_e$ CRP	Age, sex adjusted P value
Male	-0.2314	<0.001
Age (years)	-0.0600	0.006
Duration of diabetes (years)	-0.0642	0.015
HbA1c (% / mmol/mol)	0.0623	0.014
BMI (kg/m^2)	0.2429	<0.001
Systolic blood pressure (mm Hg)	0.0178	0.113
Diastolic blood pressure (mm Hg)	0.0178	0.051
Risk Factors (%)		
Current smoker	0.0585	0.003
Hypertension	0.0210	0.312
Albuminuria	-0.0037	0.524
Retinopathy	-0.0979	<0.001
Lipids		
LDL cholesterol (mmol/l)	0.0431	0.343
Total cholesterol (mmol/l)	0.1122	0.001
Triacylglycerol (mmol/l)	0.1448	<0.001
HDL cholesterol (mmol/l)	-0.0447	<0.001
Apolipoprotein A-1 (g/l)	-0.0569	<0.001
Apolipoprotein B (g/l)	0.1500	<0.001
Non-HDL cholesterol (mmol/l)	0.1305	<0.001
Total Chol/HDL ratio	0.1096	<0.001
Medication		
Insulin or oral hypoglycaemic drugs	0.0064	0.701
Blood pressure lowering drugs	0.0678	0.005
Aspirin or other antiplatelet drugs	0.0148	0.206
Oral contraceptives or HRT	0.1703	<0.001

HbA1c, glycated haemoglobin. BMI, body mass index. LDL, low-density lipoprotein.
HDL, high-density lipoprotein. Chol, cholesterol. HRT, hormone replacement therapy.
